# Supplementary material for: CDYL suppresses epileptogenesis in mice through repression of axonal Nav1.6 sodium channel expression
Source: Nat Commun. 2017 Aug 25;8:355. doi: 10.1038/s41467-017-00368-z (PMC5572458; doi:10.1038/s41467-017-00368-z)
Supplement: Supplementary file 1 — Supplementary Information [file 41467_2017_368_MOESM1_ESM.pdf]

File name: Supplementary Information
Description: Supplementary Figures and Supplementary Tables

File name: Supplementary Movie 1
Description: Typical spontaneous Class III seizure. Example video and EEG recordings describing the occurrence of first spontaneous class III seizure in latent period after status epilepticus induced by kainic acid injection.

File name: Supplementary Movie 2
Description: Typical spontaneous Class V seizure. Representative video and EEG recordings demonstrating the occurrence of first spontaneous class V seizure in latent period after status epilepticus induced by kainic acid injection.

File name: Peer Review File
Description:

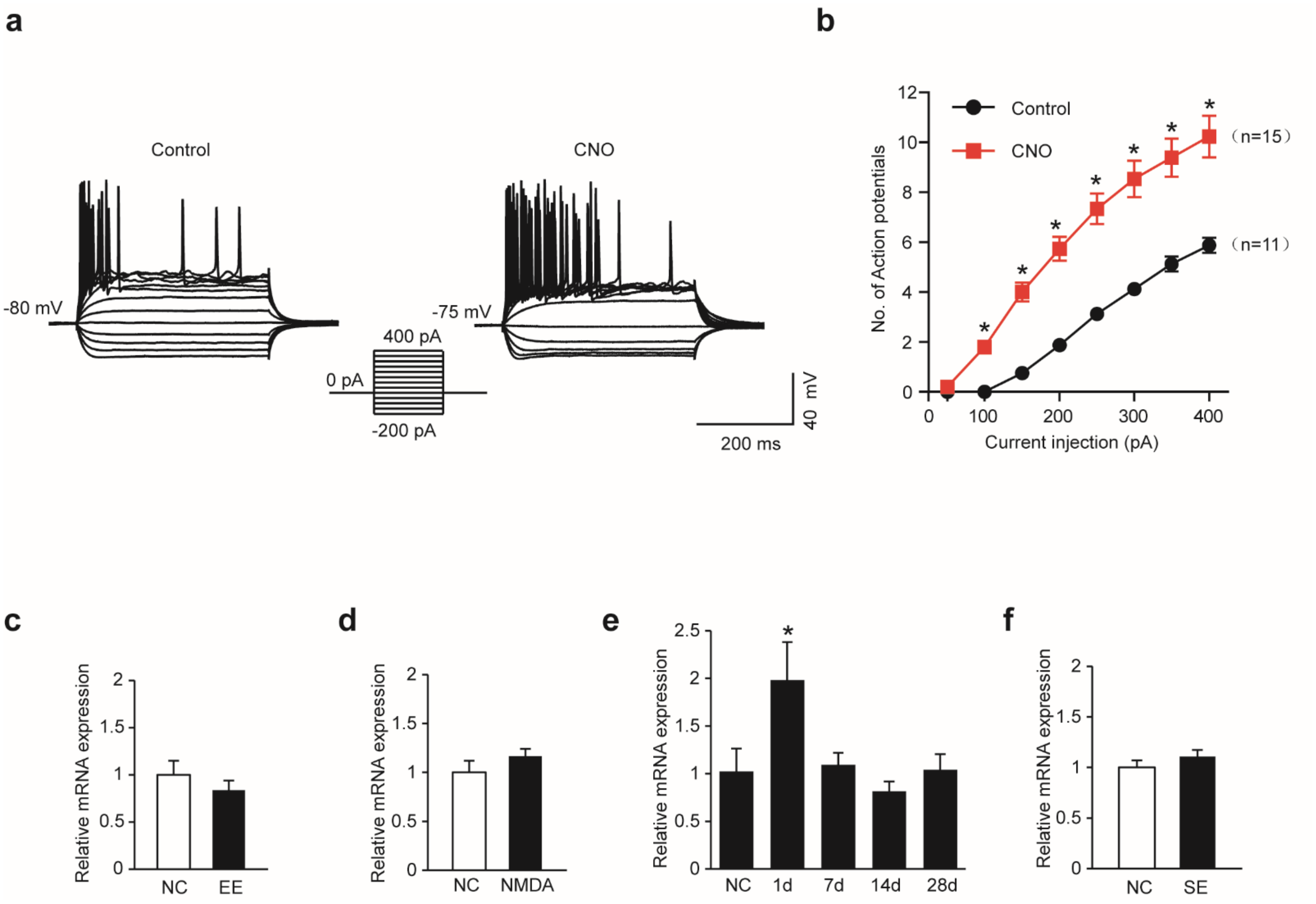

Supplementary Figure 1. The mRNA level of CDYL was not significantly changed in response to altered neuronal activity.

(a) Representative current-clamp recordings obtained from mouse DG neurons in the absence or presence of CNO. A series of 400 ms hyperpolarizing and depolarizing steps in 50 pA increments were applied to produce the traces. **(b)** The mean number of action potentials generated in response of depolarizing current pulses were shown. *$p<0.05$, one-way ANOVA with Bonferroni's multiple-comparison test. **(c)** RT-qPCR analysis showed the mRNA levels of CDYL in control rats and the rats exposed to enriched environment (EE). **(d)** RT-qPCR results from control rats and rats with intracranial injection of NMDA (10 µM) into hippocampus DG area. **(e)** RT-qPCR results from rats 7, 14, 28 days after status epilepticus in temporal lobe epilepsy model. **(f)** RT-qPCR results from rats 28 days after status epilepticus in temporal lobe epilepsy model. The data were normalized against the expression of GAPDH. *$p<0.05$, paired two-tailed Student's *t* test. Data were presented as mean ± s.e.m..

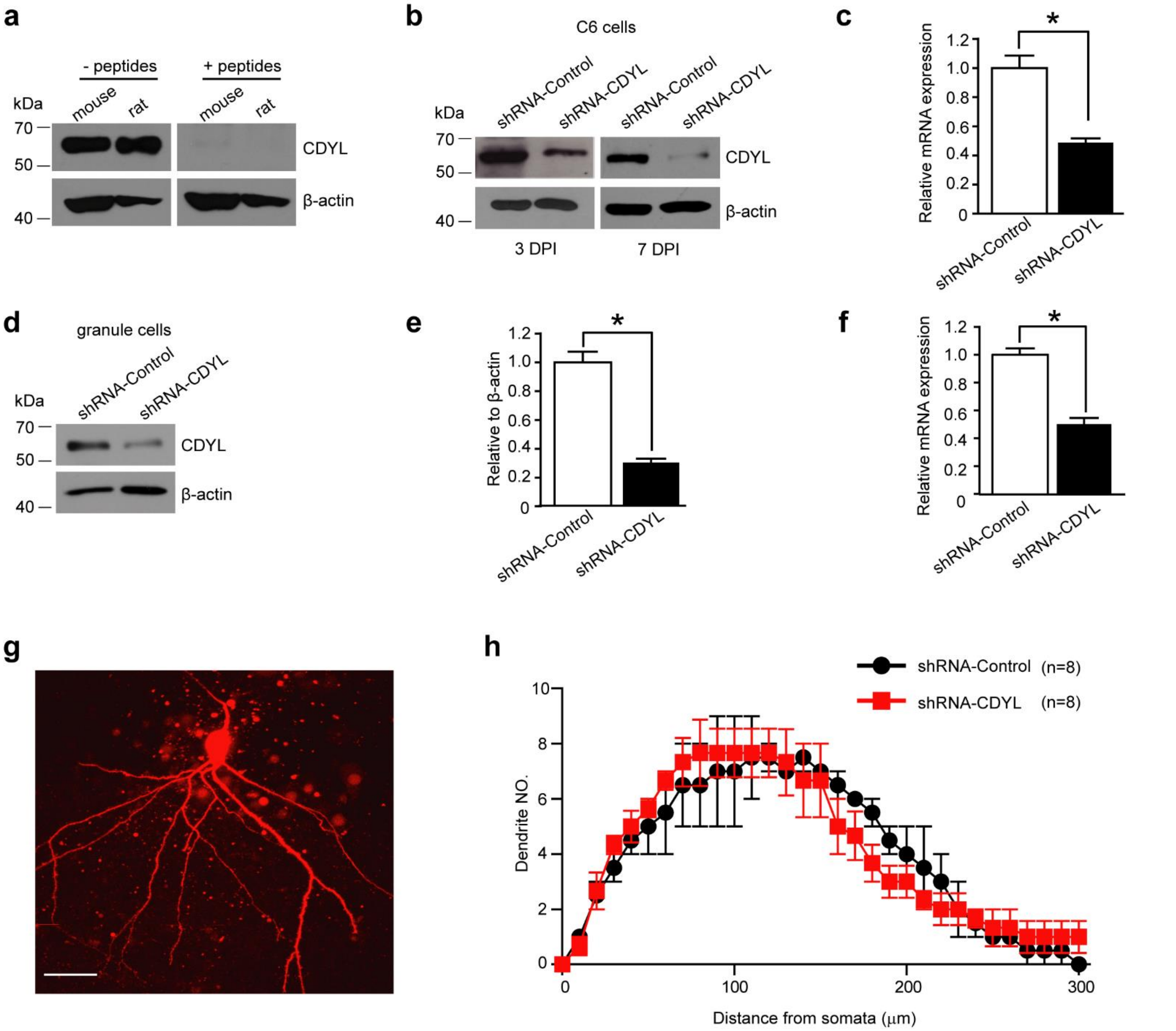

Supplementary Figure 2. Post-hoc morphological analysis of complex dendritic tree of CDYL-shRNA-infected neurons.

(a) Peptide competition assay showing CDYL antibody that we generated was specific to detect mouse and rat CDYL protein in Western blotting. The two CDYL antigenic peptides, DGFQGESPEKLDPVDQG and IDDRRDQPFDKRLRFSV, were used for this assay. **(b)** Western blotting showing CDYL was efficiently knockdown after lentivirus infection of rat C6 cells for 3 days or 7 days. β-actin was served as a control. **(c)** RT-PCR results of the lentivirus-infected C6 cells. *p<0.05, paired two-tailed Student's *t* test. **(d)** Western blotting showing CDYL was significantly reduced after CDYL-shRNA lentivirus injection into rat hippocampal DG area for 14 days. **(e)** Quantification was done by normalizing the level of CDYL to that of β-actin. **(f)** RT-PCR results showing CDYL-shRNA lentivirus injection significantly decreased CDYL mRNA level in granule cells of rat hippocampus. *p<0.05, paired two-tailed Student's *t* test. **(g)** Representative morphological images of nonsilencing-shRNA-infected rat DG neurons. Scale bar, 20 μm. **(h)** Sholl analysis of CDYL-shRNA-infected and nonsilencing-shRNA-infected rat DG neurons. Data were presented as mean ± s.e.m..

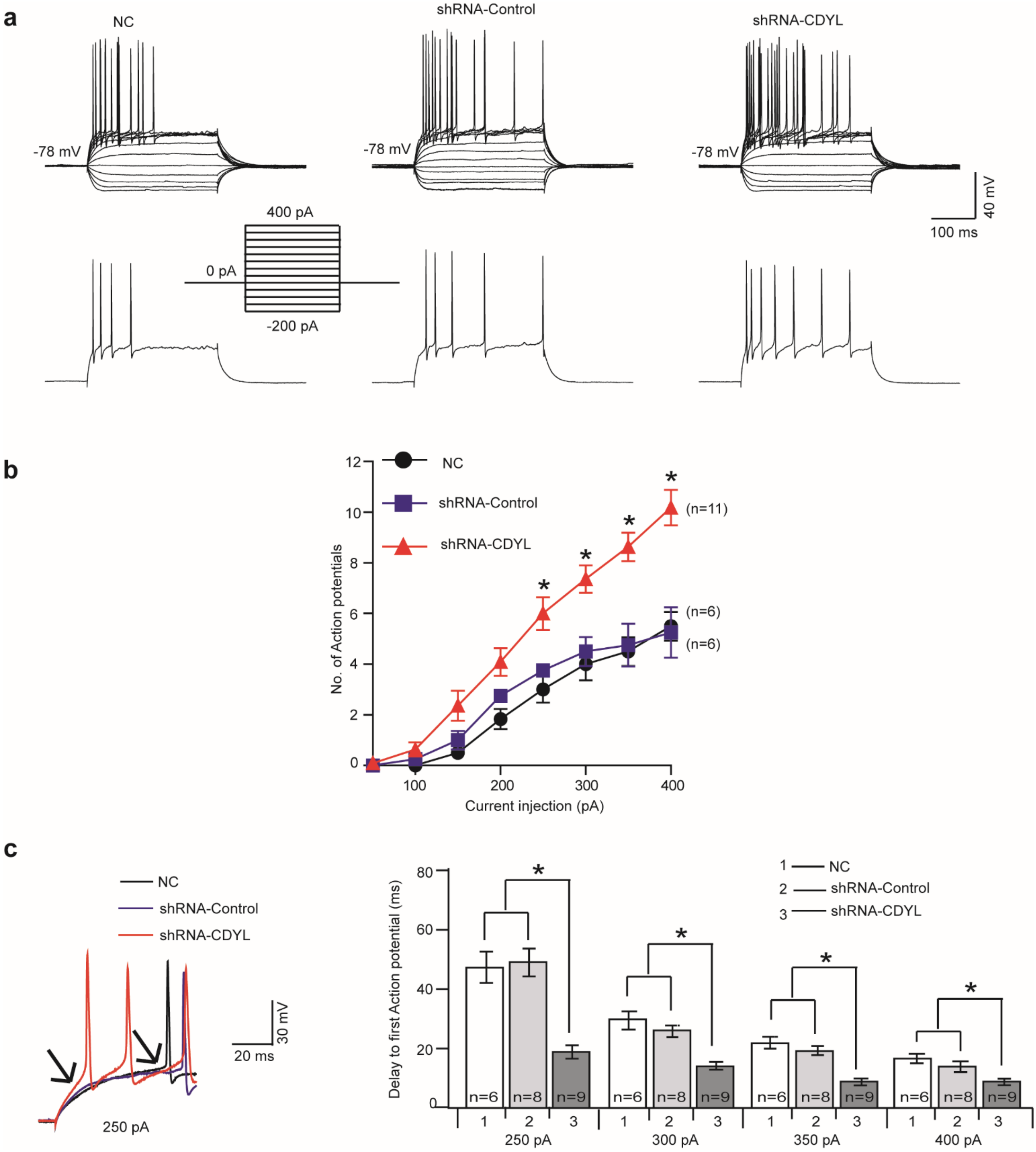

Supplementary Figure 3. The excitability of CDYL-shRNA-infected DG neurons at resting membrane potential is enhanced.

(a) Example current-clamp recordings obtained from normal control (NC), CDYL-shRNA-infected and nonsilencing-shRNA-infected rat DG neurons at resting membrane potential. A series of 400 ms hyperpolarizing and depolarizing steps in 50 pA increments were applied to produce the traces. **(b)** The mean number of action potentials generated in response of depolarizing current pulses. *$p<0.05$, one-way ANOVA with Bonferroni's multiple-comparison test. **(c)** Representative recordings in response to a series of 400 ms depolarizing at 250 pA (left). The mean time taken to first action potential in response of depolarizing current pulses at 250 pA, 300 pA, 350 pA, 400 pA, respectively (right). *$p<0.05$, one-way ANOVA with Bonferroni's multiple-comparison test. Data were presented as mean ± s.e.m..

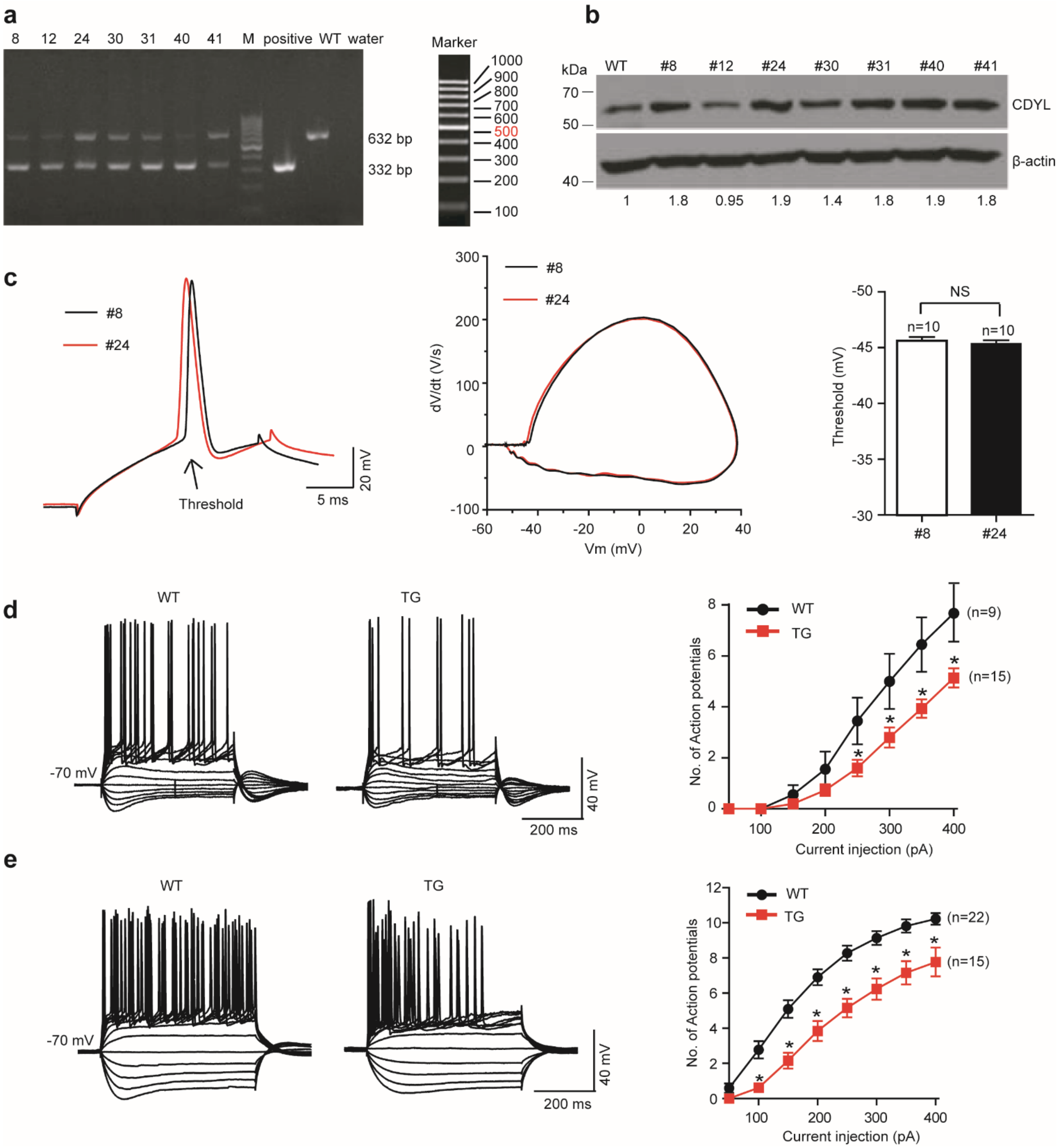

Supplementary Figure 4. The excitability of stellate cells at layer II of medial entorhinal cortex and pyramidal cells of CA1 in hippocampus of transgenic mice over-expressing CDYL is reduced.

(a) Initial identification of founders of transgenic mice over-expressing CDYL by PCR analysis of genomic DNA from mice tails. The 332 bp band is specific for transgenic mice over-expressing CDYL. Out of 47 pups screened, 7 pups were identified positive. **(b)** Examination of CDYL protein expression in the transgenic founders by western blot analysis. Quantification was done by ImageJ (bottom). **(c)** Typical spikes (left), associated phase plane plots (middle), and the average spike threshold (right) obtained from DG neurons of progenies from founders #8 and #24 of transgenic mice over-expressing CDYL. **(d-e)** Representative traces obtained from cortex **(d)** and CA1 neurons **(e)** from WT and CDYL-TG mice in response to a series of 400 ms current stepping from -200 to +400 pA with increments of 50 pA. For comparison reasons, the recordings were obtained at the fixed potential of -70 mV (left). Graphs to demonstrate average numbers of action potentials were obtained in response to varying depolarizing current pulses (right). *$p<0.05$, one-way ANOVA with Bonferroni's multiple-comparison test. Data were presented as mean ± s.e.m..

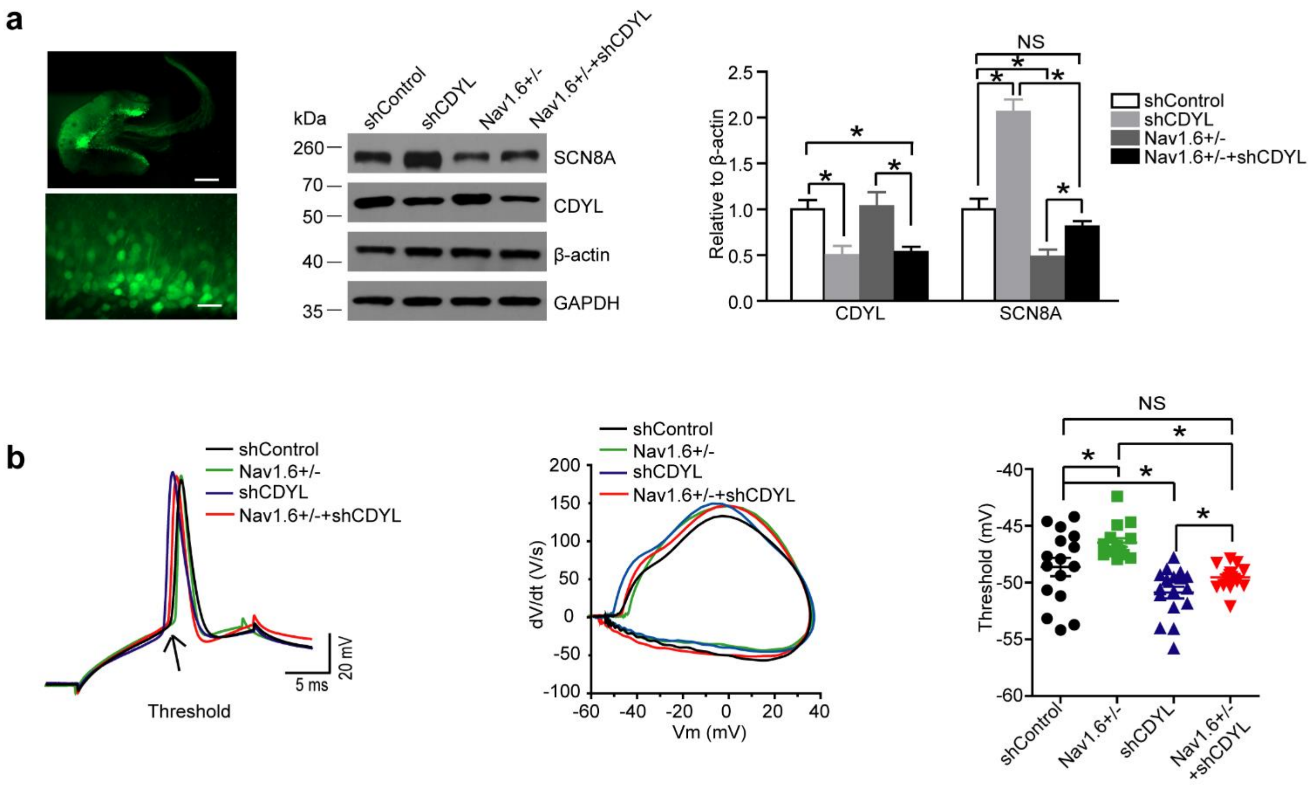

Supplementary Figure 5. CDYL mediated neuronal intrinsic excitability through Nav1.6 ion channel. **(a)** Expression of CDYL-shRNA lentivirus in 14 day after infecting the dorsal hippocampal DG area of SCN8A null heterozygote mice (left). Scale bar, 200 μm (upper); 20 μm (bottom). Knockdown of CDYL increased SCN8A expression. Western blot analysis (middle) and quantification (right) were performed. Hippocampal DG tissues were obtained from Control-shRNA-infected, CDYL-shRNA-infected, SCN8A null heterozygote mice and SCN8A null heterozygote mice infected CDYL-shRNA lentivirus. n=3, *p<0.05, one-way ANOVA with Bonferroni's multiple-comparison test. **(b)** Typical spikes (left), associated phase plane plots (middle), and the average spike threshold (right) obtained from DG neurons of Control-shRNA-infected, SCN8A null heterozygote, CDYL-shRNA-infected and CDYL-shRNA-infected in SCN8A null heterozygote mice. *p<0.05, one-way ANOVA with Bonferroni's multiple-comparison test. Data were presented as mean ± s.e.m..

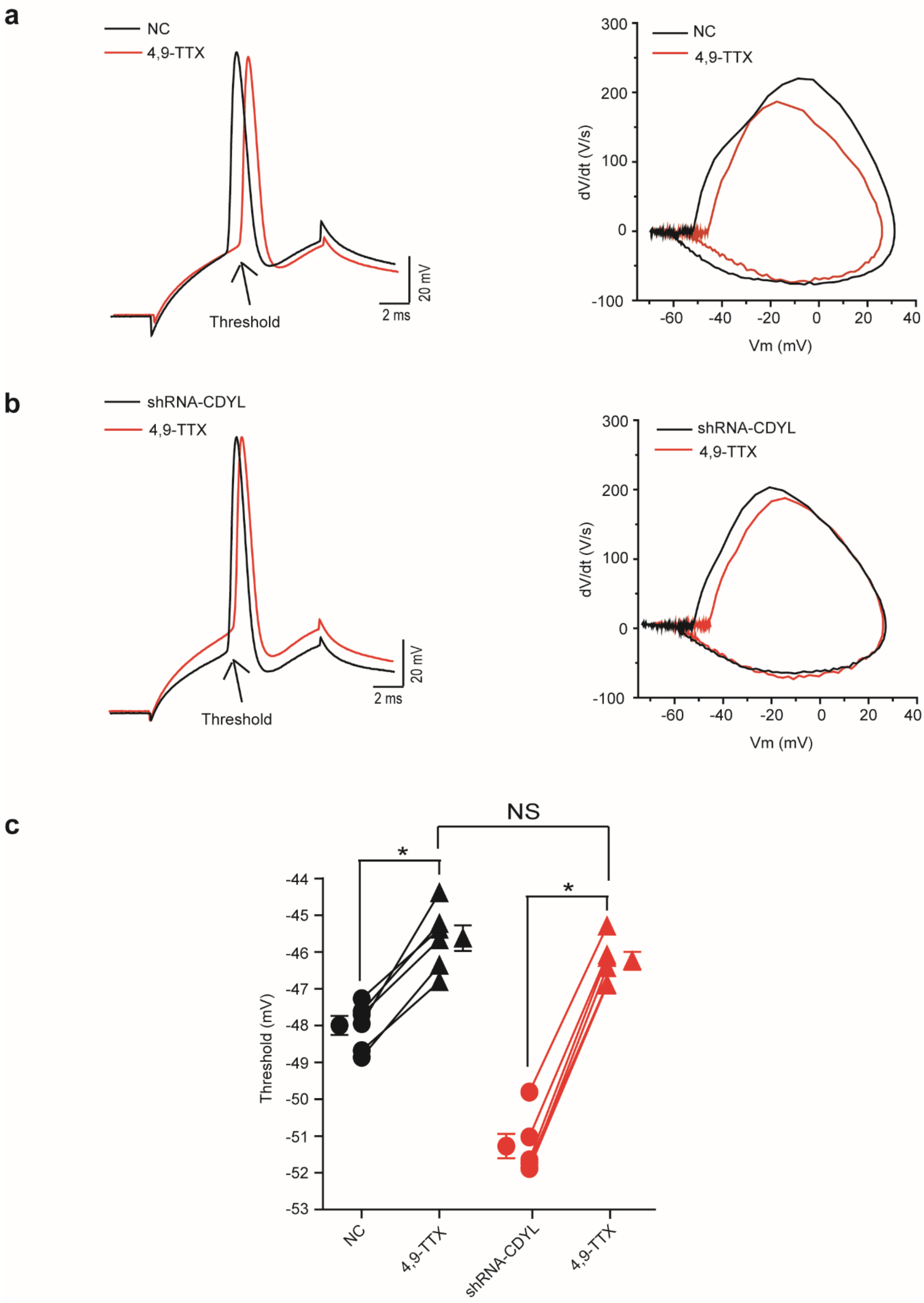

Supplementary Figure 6. Knockdown of CDYL does not affect AP threshold in the presence of 4,9-TTX. Representative action potentials and associated phase plane plots obtained from NC **(a)** and CDYL-shRNA-infected **(b)** rat DG neurons in the presence or absence of 75 nM 4,9-TTX. **(c)** Graphs showing the average AP threshold before and after adding 75 nM 4,9-TTX. *p<0.05, one-way ANOVA with Bonferroni's multiple-comparison test. Data were presented as mean ± s.e.m..

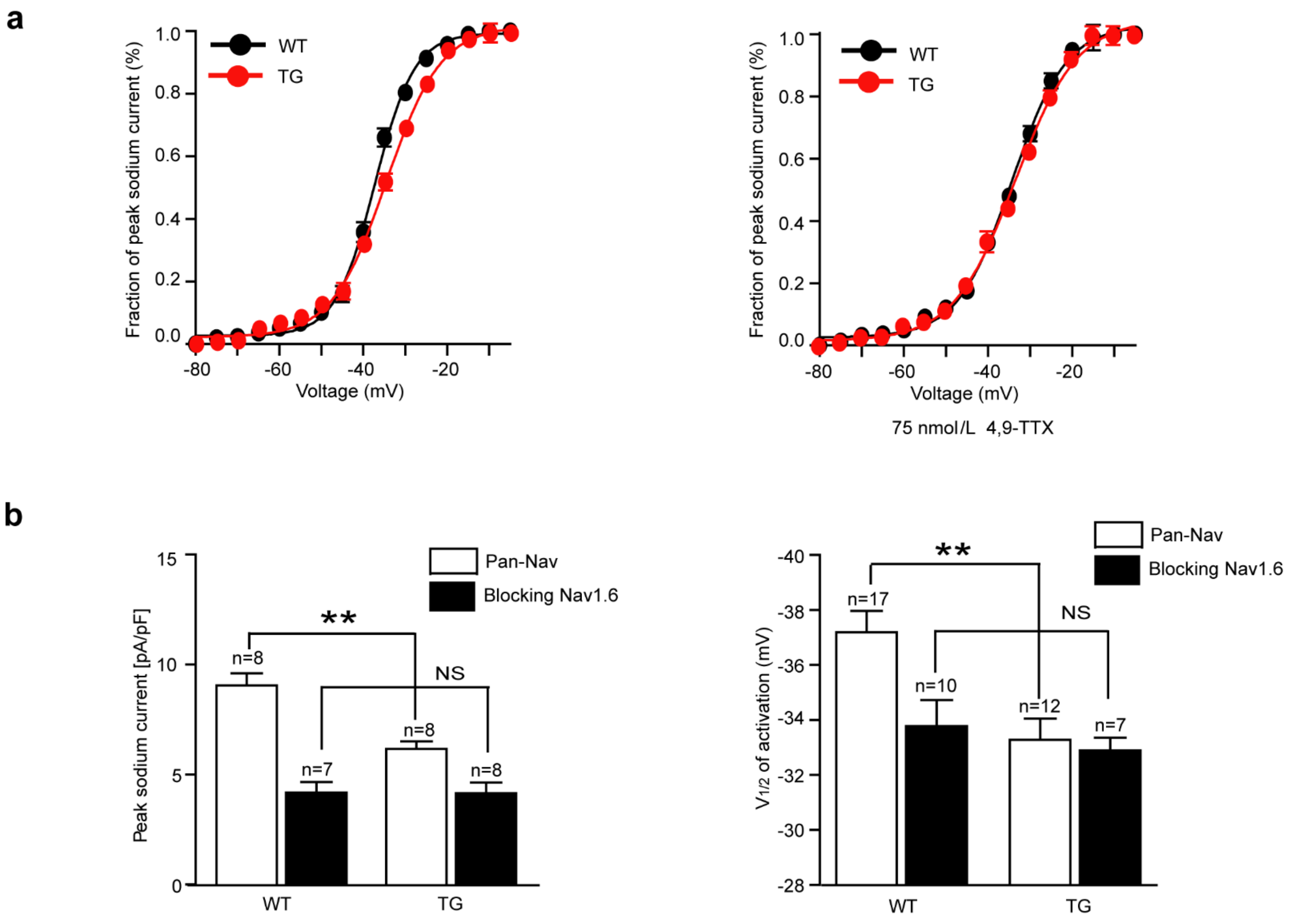

Supplementary Figure 7. Sodium currents were compromised in CDYL transgenic neurons.
(a) Graphs showing the activation curves obtained from WT and CDYL-TG mice DG neurons in the presence or absence of 75 nM 4,9-TTX, respectively. **(b)** Graphs showing the density of peak sodium currents (left) and half-activation values of sodium currents (right), respectively. **p<0.01, unpaired two-tailed Student's *t* test. Data were presented as mean ± s.e.m..

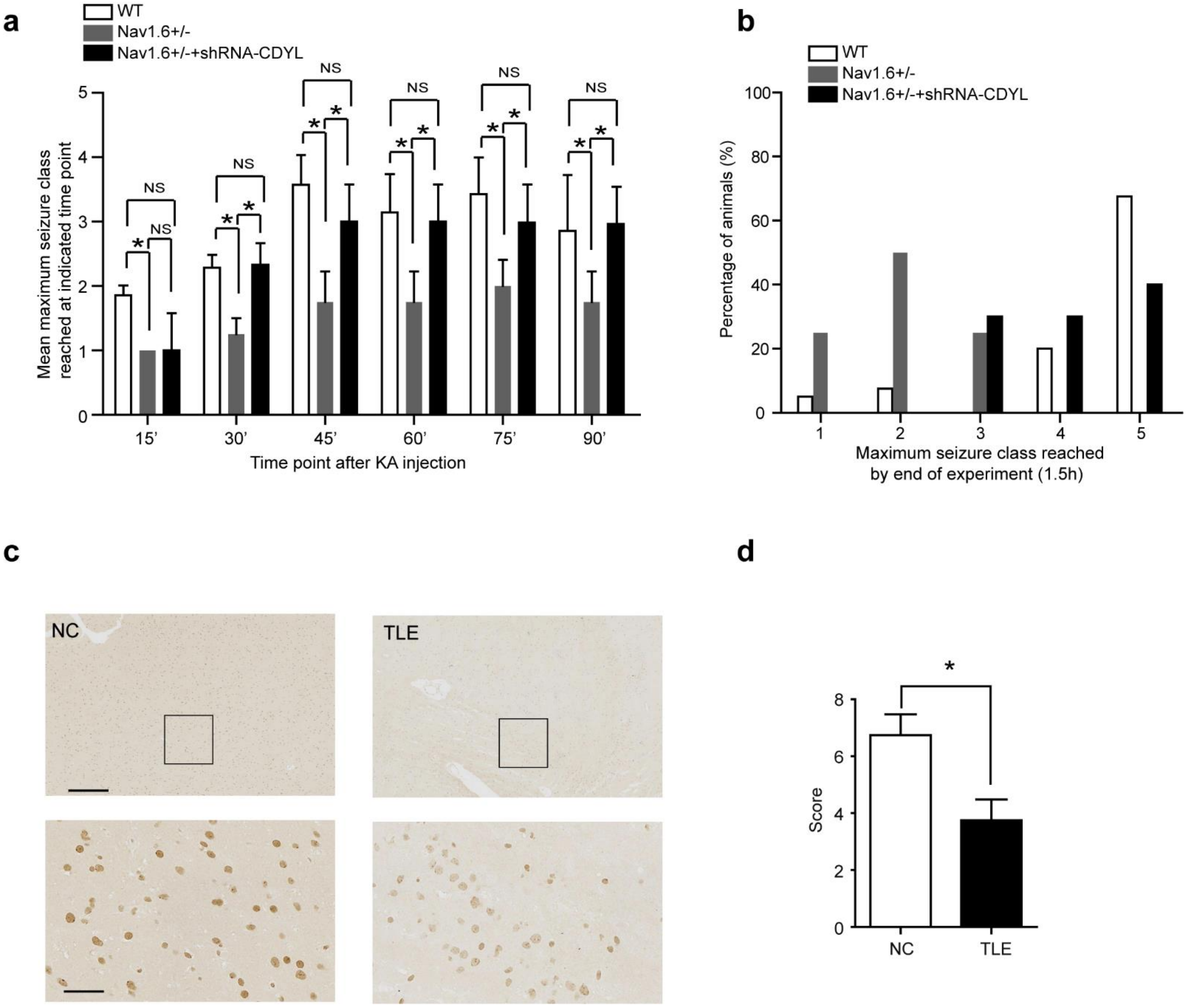

Supplementary Figure 8. CDYL protein is decreased in the neuron of epileptic foci in human intractable temporal lobe epilepsy samples.

(a) Graph depicting the seizure progression in WT, SCN8A null heterozygotes mice and CDYL-shRNA-infected in SCN8A null heterozygote mice, illustrated as mean maximum seizure class reached by 15, 30, 45, 60, 75, 90 min after KA administration. *$p<0.05$, one-way ANOVA with Bonferroni's multiple-comparison test. **(b)** Incidence of maximum seizure class reached during the course of the experiments in **(a)**. **(c)** Immunohistochemistry (IHC) results showing CDYL protein was decreased in the neurons of epileptic foci in human intractable temporal lobe epilepsy (TLE) samples. Scale bar, 400 μm (upper); 40 μm (bottom). **(d)** Quantification of CDYL protein levels in the adjacent normal tissues (NC) and hippocampus obtained from human TLE samples. n=4, *$p<0.05$, paired two-tailed Student's *t* test. Data were presented as mean ± s.e.m..

Uncropped blots related to **Fig. 1b**

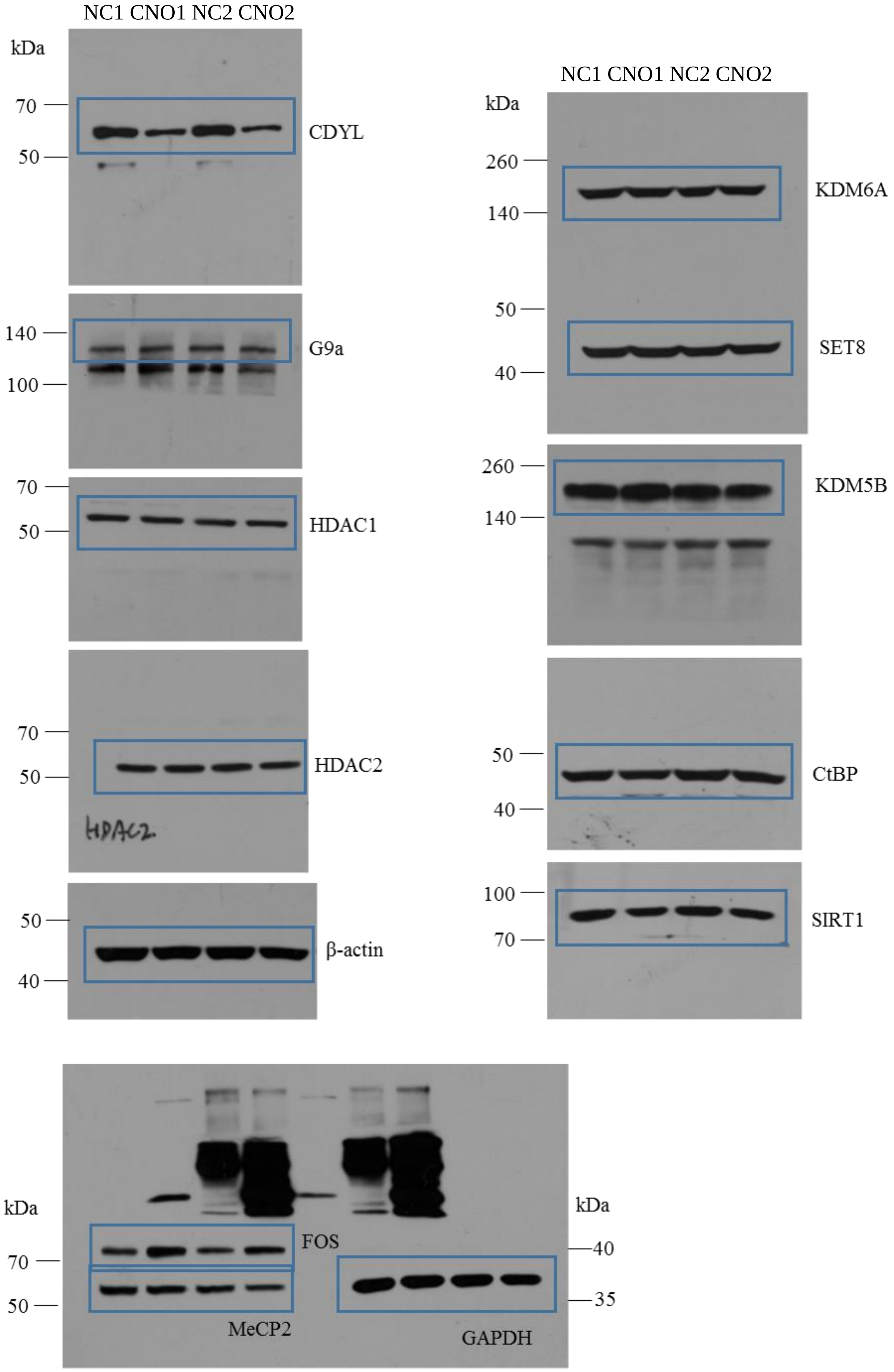

Uncropped blots related to Fig. **1e**

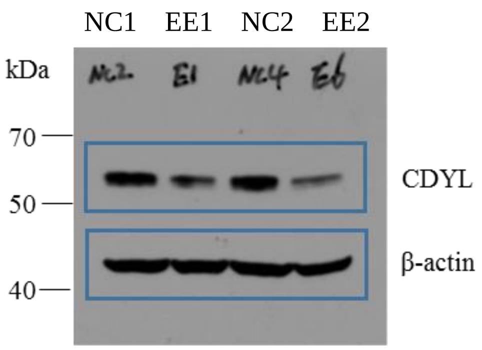

Uncropped blots related to Fig. **1f**

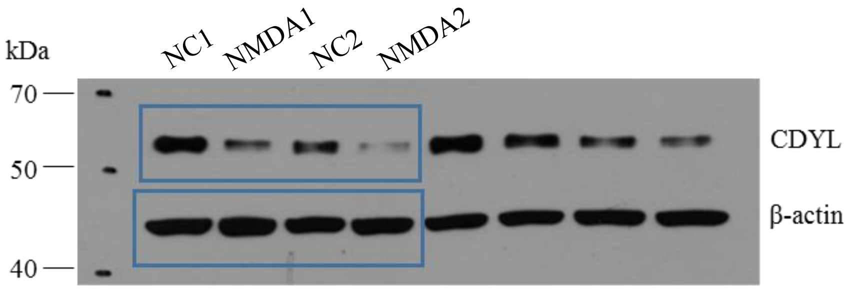

Uncropped blots related to Fig. **1g**

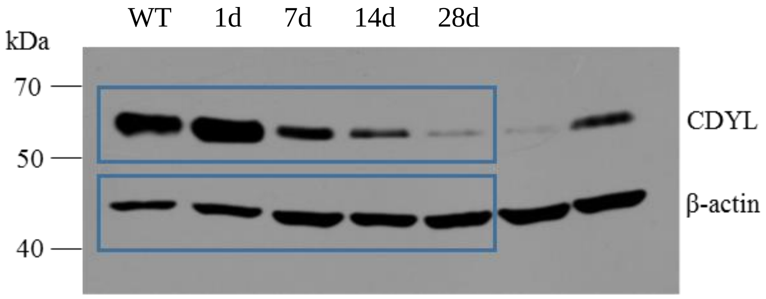

Uncropped blots related to Fig. **1h**

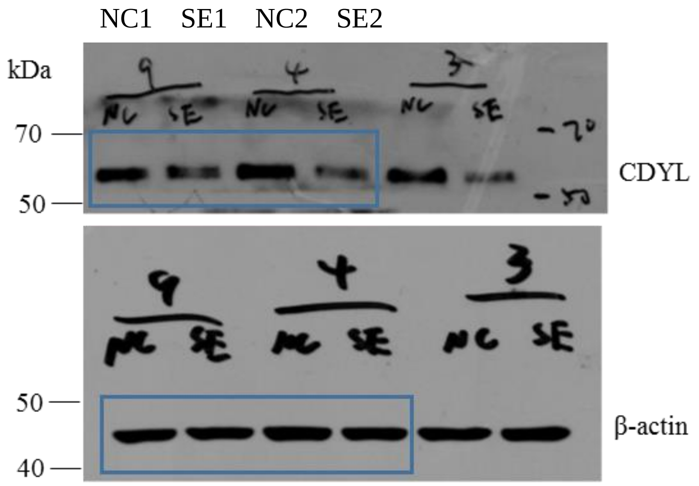

Uncropped blots related to Fig. **3a**

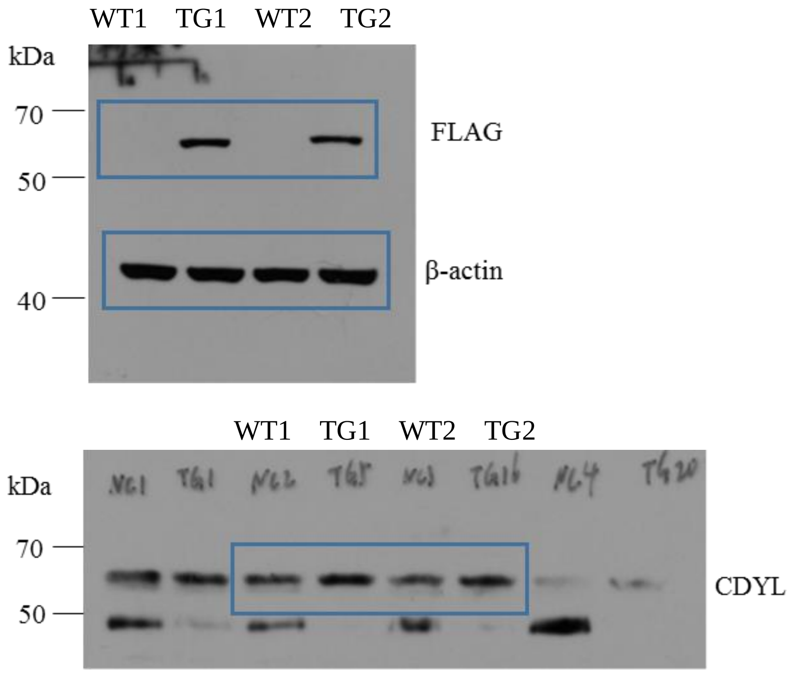

Uncropped blots related to Fig. **5a**

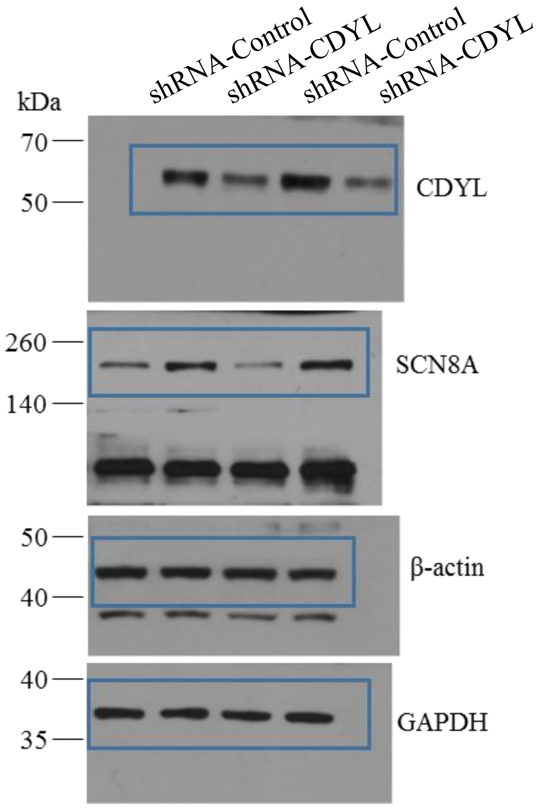

Uncropped blots related to Fig. **5b**

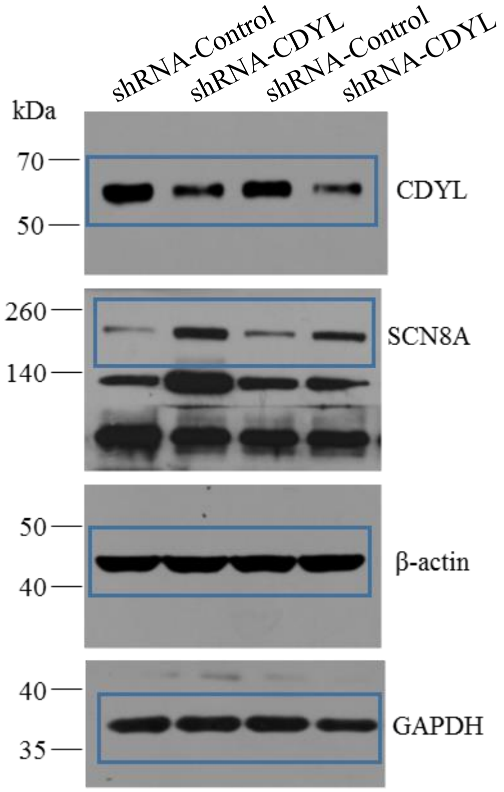

Uncropped blots related to Fig. **5c**

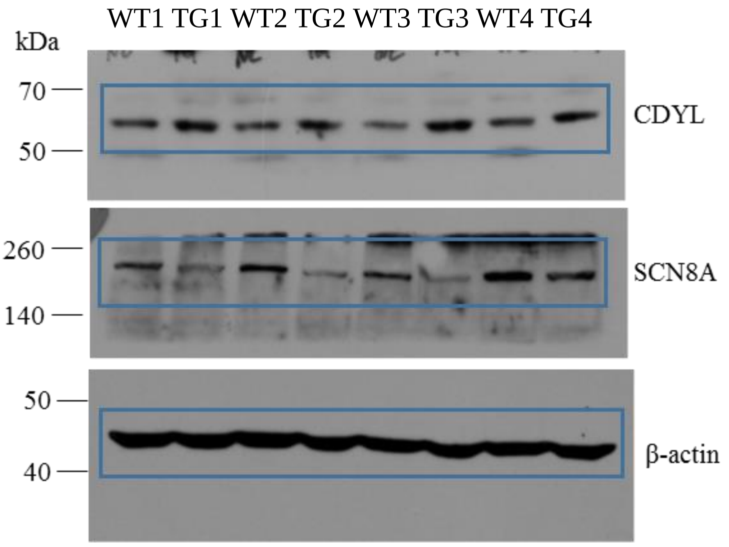

Uncropped blots related to Fig. **5e**

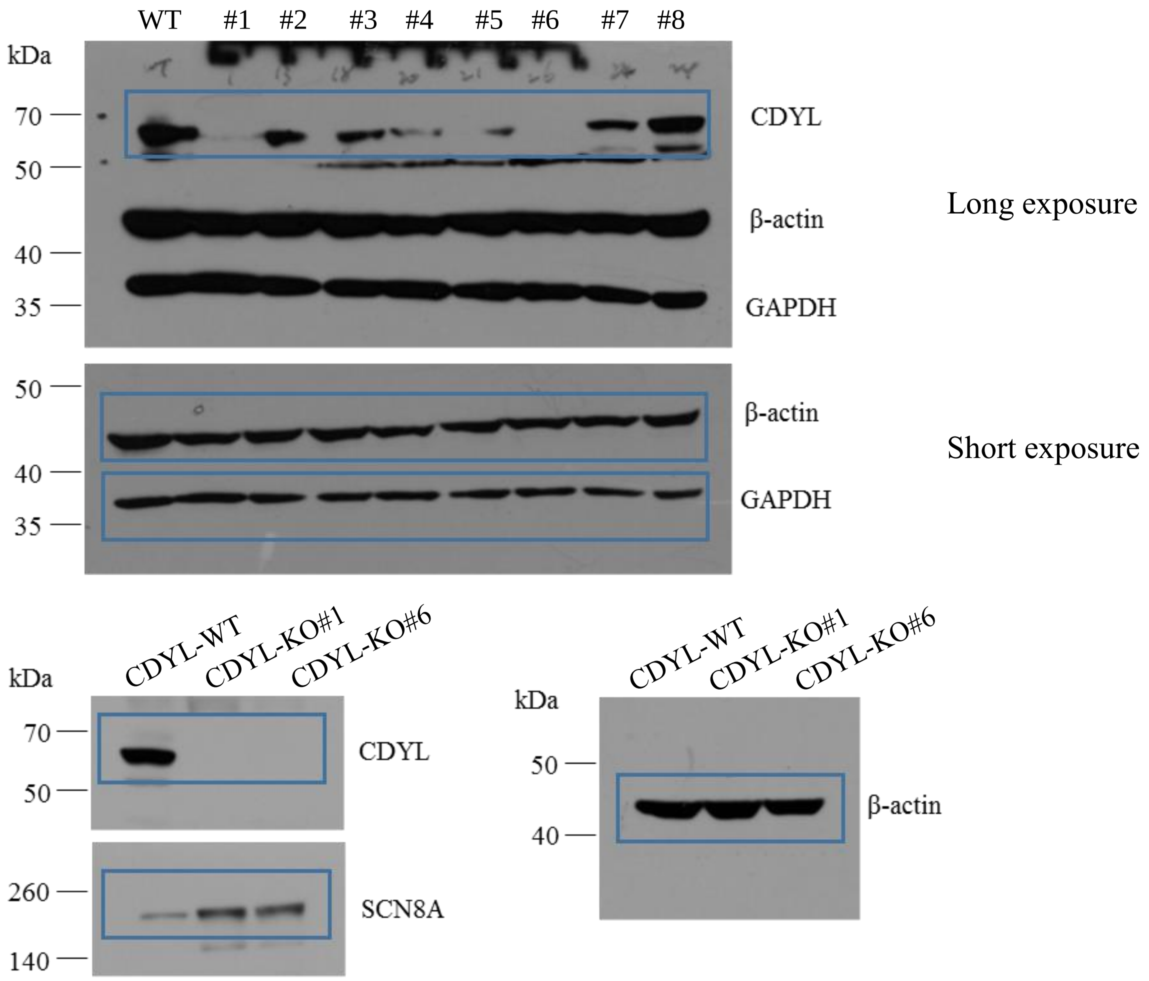

Uncropped gels related to Fig. **5f**

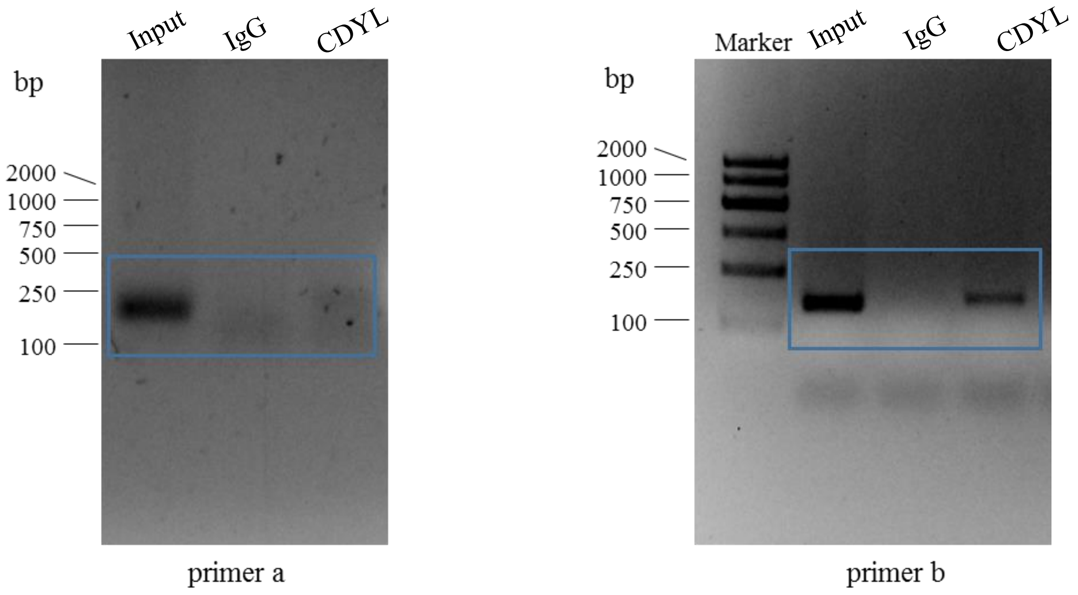

Uncropped blots related to Fig. **5h**

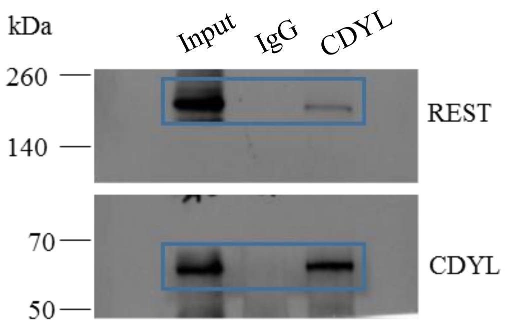

Uncropped blots related to Fig. **5i**

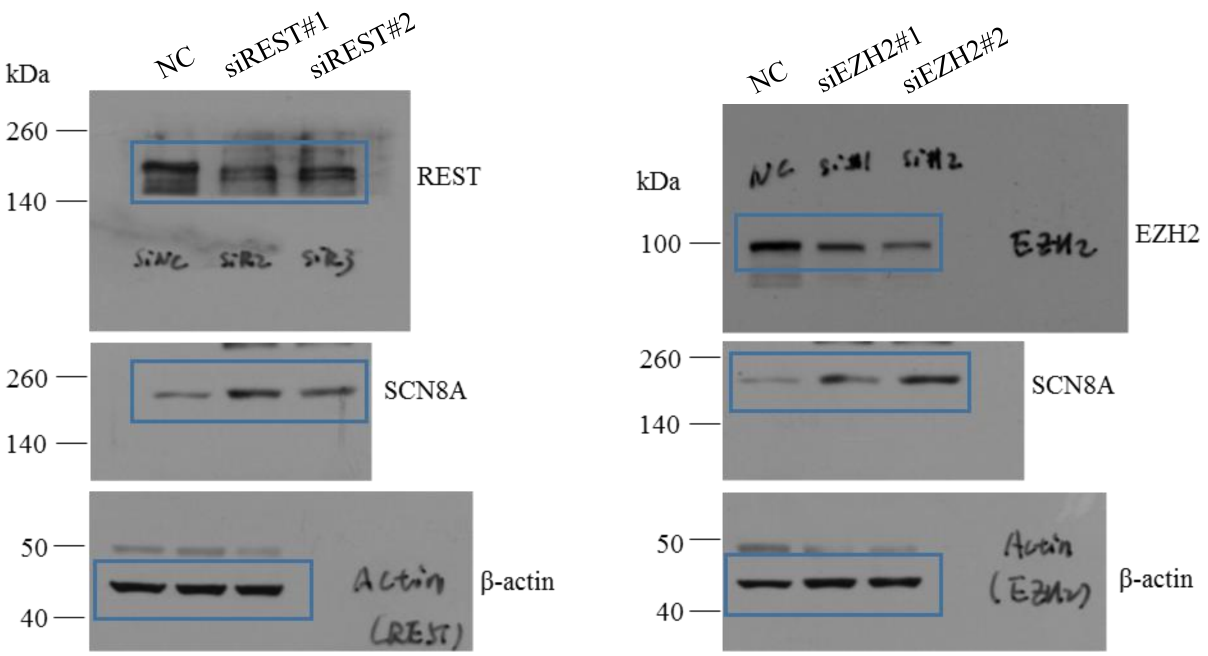

Uncropped blots related to Fig. **6c**

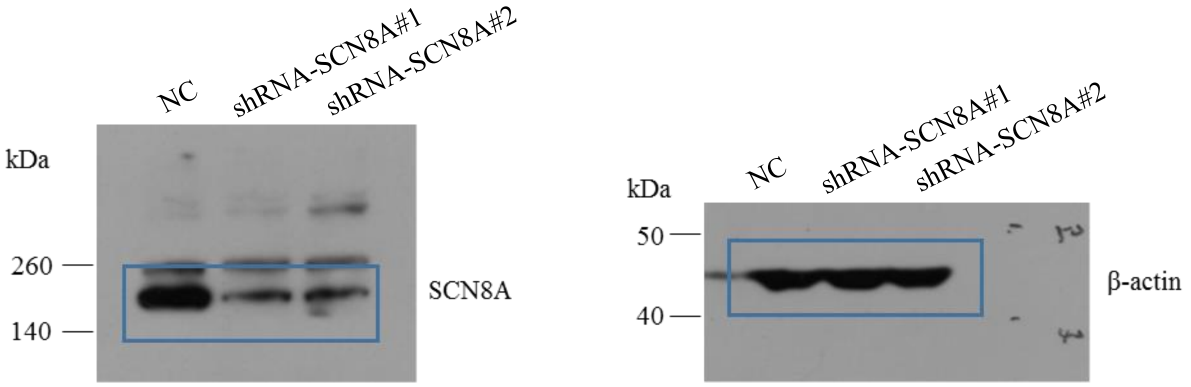

Uncropped blots related to Fig. **8j**

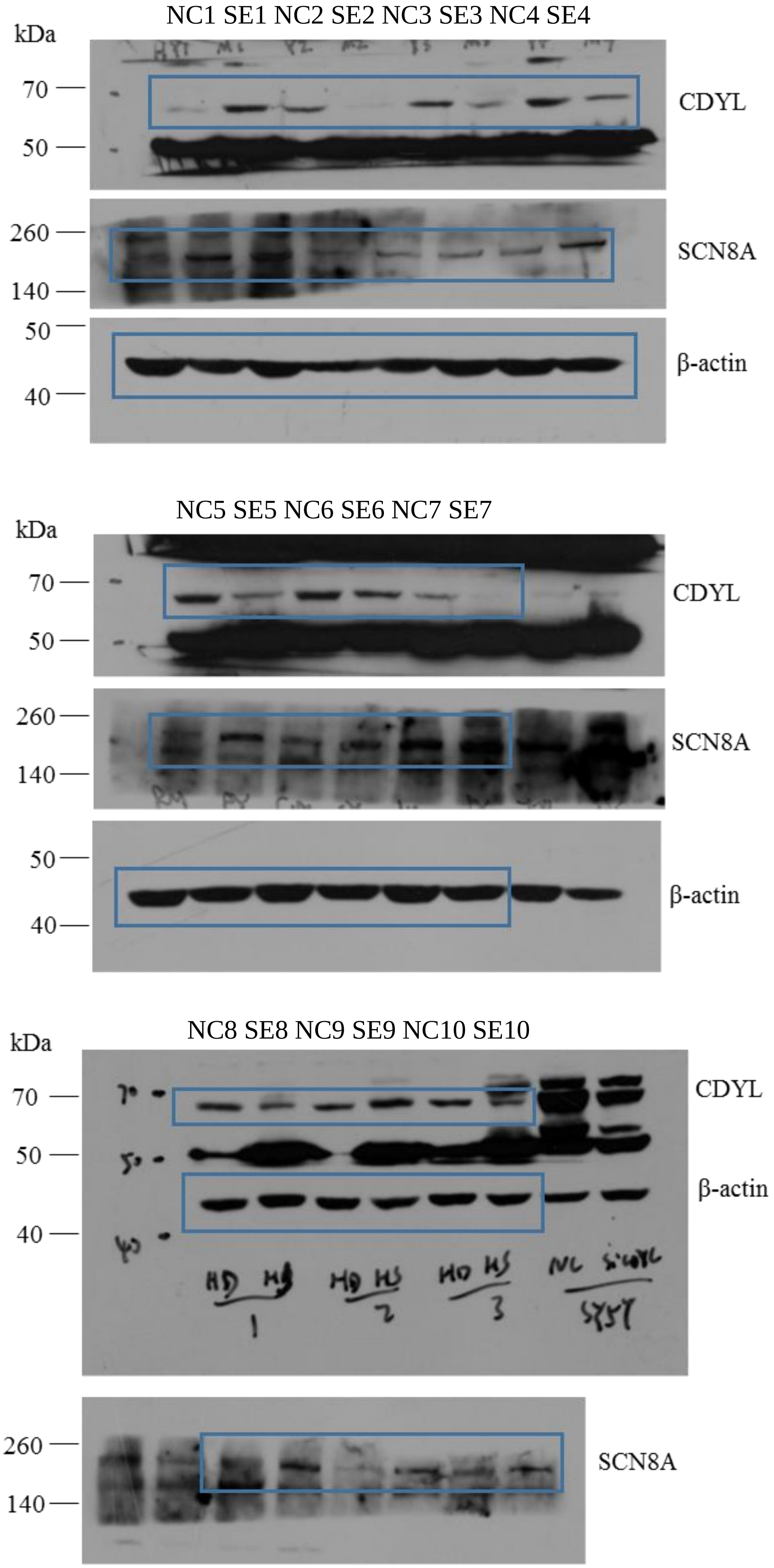

Supplementary Figure 9. Uncropped blots and gels to Fig 1-8.

The relevant figures are indicated in the blot titles. In some blots, both lower and higher exposed ones with markers are provided. The cropped areas within the blue boxes are indicated.

Supplementary Table 1. Electrophysiological properties of hippocampal DG neurons of rats

Parameters	Non-infected (n=33)	nonsilencing-shRNA -infected (n=20)	shRNA-CDYL -infected (n=34)
Resting potential (mV)	-78.08±0.48	-77.23±0.89	-77.91±0.58
Input resistance (MΩ)	166.38±6.11	169.42±19.25	178.59±11.83
AP Threshold (mV)	-48.01±0.26	-47.51±0.53	-51.41±0.36*
AP Amplitude (mV)	83.03±1.58	83.71±2.21	87.37±1.91*

Data are reported as mean ± s.e.m. for cells of hippocampal dentate gyrus (DG) of rats. *p<0.05, one-way ANOVA with Bonferroni' s multiple-comparison test.

Supplementary Table 2. Electrophysiological properties of hippocampal DG neurons of mice

Parameters	Wide-type (n=31)	Transgenic mice over-expressing CDYL (n=34)
Resting potential (mV)	-78.72±0.33	-79.04±0.41
Input resistance (MΩ)	166.66±12.64	149.19±7.72
AP Threshold (mV)	-48.65±0.37	-45.03±0.51*
AP Amplitude (mV)	83.86±0.65	79.96±1.45*

Data are reported as mean ± s.e.m. for cells of hippocampal dentate gyrus (DG) of mice. *p<0.05, one-way ANOVA with Bonferroni' s multiple-comparison test.

Supplementary Table 3. Demographic and clinical characteristics of selected patients with mesial TLE

Patients* (Number)	Age (Years)	Sex	Duration of disease (Years)	Medication history	MRI examination	Epileptic foci (Named SE1-14)	Type of surgery
1 (SE1/NC1)	57	Male	4	Carbamazepine, Topiramate	Normal	Seizure on-set tissues from hippocampus confirmed by scalp and intracranial EEG	Anterior temporal lobe and Hippocampus -Amygdala resection
2 (SE2/NC2)	30	Male	12	Valporate	L hippocampal atrophy, no signal changes		
3 (SE3/NC3)	27	Male	9	Valporate	R hippocampal atrophy, no signal changes		
4 (SE4/NC4)	26	Male	18	Carbamazepine	L temporal-lobe atrophy, no signal changes		
5 (SE5/NC5)	29	Male	5	Lamotrigine, Gabapentin	Normal		
6 (SE6/NC6)	50	Female	20	Valporate	Normal		
7 (SE7/NC7)	42	Female	31	Carbamazepine, Phenobarbital	L temporal-lobe and hippocampal atrophy, no signal changes		
8 (SE8/NC8)	29	Male	2	Valporate	Normal		
9 (SE9/NC9)	15	Female	11	Phenobarbital	Normal		
10 (SE10/NC10)	29	Male	8	Phenytoin, Lamotrigine	Normal		
11 (SE11/NC11)	51	Female	2	Phenobarbital	Normal		
12 (SE12/NC12)	35	Female	11	Lamotrigine, Phenobarbital, Phenytoin	R hippocampal atrophy, no signal changes		
13 (SE13/NC13)	39	Female	9	Carbamazepine, Phenobarbital	Normal		
14 (SE14/NC14)	27	Male	3	Carbamazepine, Valporate	Normal		

*All selected patients were seizure-free for periods of 2 or 3 years.

Supplementary Table 4. qRT-PCR primers

Gene	Strand	Sequence
human CDYL	F	CGAGGAGCTGTACGAGGTTG
human CDYL	R	ACGAGTGCCTTAGGAGAGGT
human SCN8A	F	AGCTGACATGGTTGCTGGTT
human SCN8A	R	TCCAGTCAACTTCTGACGCC
human GAPDH	F	CTCCTGTTCGACAGTCAGCC
human GAPDH	R	TGGAATTTGCCATGGGTGGA
rat CDYL	F	CCTCTTTGTGATGTGGTTTGG
rat CDYL	R	CATCTCATTTGCAGACGCTC
rat SCN8A	F	GGCAAACATCGAGAGGCGTA
rat SCN8A	R	CACTGTTTGGCTTGGGCTTG
rat GAPDH	F	ATCACTGCCACTCAGAAGAC
rat GAPDH	R	CATCATACTTGGCAGGTTTCTC
mouse CDYL	F	GCCTAATTCACAGGAAGCCCA
mouse CDYL	R	TTGAATTGCAGGGGACGCTT
mouse SCN8A	F	GGCAATGTTTCAGCTCTACGC
mouse SCN8A	R	TTGAATTGCAGGGGACGCTT
mouse RIMS2	F	GAGCTGGAAAGCGAGAGTGT
mouse RIMS2	R	CCGACAAAACTCCCTGCTCA
mouse BDNF	F	TGACAAGGCGAAGGGTTTCT
mouse BDNF	R	CAATTTGCACGCCGATCCTT
mouse GABRB3	F	AGGAAGGCTTTTCGGCATCT
mouse GABRB3	R	ATGTTCCCGGGGTCGTTTAC

mouse CHRM4	F	CTCTGGCTAGTTCCGCCGTC
mouse CHRM4	R	TTGTGGGCTGTTGTGACCAG
mouse GLRA1	F	TTGCCTGCTCTTCGTGTTCT
mouse GLRA1	R	GCATGGGGCTCTTGTGATGT
mouse VGF	F	CCCTCGACCATCGCTCATAC
mouse VGF	R	GGTTTTCATGACCAACGGGC
mouse CACNG3	F	CCCTCATCCCCCAGACCTTA
mouse CACNG3	R	CCGATGCTATGACTGCACCT
mouse NPAS4	F	TTCCCCTACGAAGGGTTTGC
mouse NPAS4	R	CTGGGATCGCTCGAAGTCTC
mouse SCNN1G	F	GCCGTGACCCTTCAGTTCAG
mouse SCNN1G	R	CTTAATGGTCGGTGCCTGGG
mouse GAPDH	F	CCCTTAAGAGGGATGCTGCC
mouse GAPDH	R	ACTGTGCCGTTGAATTTGCC

Supplementary Table 5. ChIP and qChIP primers

**Gene**	**Strand**	**Sequence**
mouse SCN8A	F	TCACTGGTTTCTTGGGGTCG
mouse SCN8A	R	TCTAGCAAGCGTAACGCCAA
mouse RIMS2	F	GTCAGCCCATTCACACTCCT
mouse RIMS2	R	TCGTCTCCCTGGACAAGACA
mouse BDNF	F	TTCAGCACCTTGGACAGAGC

mouse BDNF	R	TGGCTTGACAGCGAGGAAA
mouse GABRB3	F	TCCTTCAGCGTAAACGACCC
mouse GABRB3	R	TCTCAGGCGAATGTCGTAGC
mouse CHRM4	F	CAGCCCTTGCAGGAGAGTAG
mouse CHRM4	R	GAGTACCGGTCACCCACAAA
mouse GLRA1	F	TCCTGAACCCCTATTGCACC
mouse GLRA1	R	CCCCCATAACTCGTGGACTT
mouse VGF	F	TTTATAAGGCAGAGGCGGCG
mouse VGF	R	TGTTCCGTGGCTGGAGTATG
mouse CACNG3	F	GGGTGGGCTCTGAGATAACG
mouse CACNG3	R	CCAAGCAAAGTTTCGGGAGC
mouse NPAS4	F	TCCCACTCAGAATGGTCGTG
mouse NPAS4	R	GCCACACCTGTAAATAGCCCA
mouse SCNN1G	F	ACACCAGAGACTTTCAGCAACA
mouse SCNN1G	R	GTTGCCACACATGGATTGCC
mouse GRM8	F	AATCTGGGCCATTTCCCTCC
mouse GRM8	R	GGAAATGCAAGCAGATGTTGGT
mouse GAPDH	F	GTGCCTCATCCTTGCACAACTG
mouse GAPDH	R	CCCCACAAGGCAAACAGAAATAAG
SCN 8A primer a	F	GAAGTGCATGCTCCCCGTTA
SCN 8A primer a	R	ATTTCGACCGCTCACTCCAC
SCN 8A primer b	F	ACCATACGGTTGCTGTTGCT
SCN 8A primer b	R	GAACTGGTCGGGGAACTGTC

Supplementary Table 6. GO analysis of ChIP-Seq against CDYL

Category	Name	p-value	Gene number	Gene name
Pathway	gated channel activity	3.27E-06	119	SCN8A,SCN9A,SCNN1G,KCNH7,ASIC2,STIM2,ANXA6,KCNAB2,SHROOM2,AQP1,ANO8,HCN2,C15orf27,CACNA1H,CACNA1A,CACNA1B,CACNA1C,CACNA1D,CACNA1E,CACNA2D1,CACNB2,CACNB4,CHRNA7,CHRNB2,CLCN4,CLCNKB,CNGA1,CNGA3,PIEZO1,GRIN3A,KCNMB2,KCNK9,CACNG3,CACNG2,PEX5L,CACNG5,GABRA3,KCNH5,GABRA4,GABRA5,GABRB1,GABRB2,GABRB3,GABRG3,GABRR1,GABRR2,ASIC5,GLRA1,GLRA2,TMC1,TMC2,GRIA1,GRIA2,GRIA4,GRID1,GRID2,GRIK1,GRIK2,GRIK3,GRIK4,GRIN2A,GRIN2B,GRIN2C,GRM7,IL1RAPL1,KCNA5,KCNC2,KCND2,KCND3,KCNH1,KCNJ5,KCNJ6,KCNJ16,KCNK2,KCNMA1,KCNN3,KCNQ1,KCNQ3,KCNS3,CLIC5,HCN1,KCNK10,TRPM5,NALCN,TMEM38A,CNGB3,ANO1,REST,KCNIP2,KCNIP1,RYR1,RYR2,RYR3,SCN2A,SCN4B,CLCA4,CHRNA9,RASA3,PIEZO2,KCNIP4,SNAP25,ANO3,CACNA2D3,SCN3B,STIM1,ANO4,TRPC1,TRPC4,ANO5,KCNH6,KCNQ5,KCNK13,KCNK12,KCNT2,KCNAB1,ANO2,GLRA3,JPH2,ANO6
Pathway	glutamate receptor activity	2.60E-05	20	GRIN3A,GRIA1,GRIA2,GRIA4,GRID1,GRID2,GRIK1,GRIK2,GRIK3,GRIK4,GRIN2A,GRIN2B,GRIN2C,GRM1,GRM2,GRM3,GRM4,GRM5,GRM7,GRM8
Pathway	ion channel activity	5.84E-05	138	SCN8A,SCNN1G,KCNH7,ASIC2,STIM2,ANXA6,KCNAB2,SHROOM2,AQP1,AQP6,ANO8,SLC26A7,MCU,HCN2,C15orf27,CACNA1H,CACNA1A,CACNA1B,CACNA1C,CACNA1D,CACNA1E,CACNA2D1,CACNB2,CACNB4,TRPA1,CHRNA7,CHRNB2,CLCN4,CLCNKB,SLC24A2,CNGA1,CNGA3,TRPM6,PIEZO1,GRIN3A,KCNMB2,KCNK9,CACNG3,CACNG2,PEX5L,CACNG5,GABRA3,KCNH5,GABRA4,GABRA5,GABRB1,GABRB2,GABRB3,GABRG3,GABRR1,GABRR2,ASIC5,GLRA1,GLRA2,GPM6A,TMC1,TMC2,GRIA1,GRIA2,GRIA4,GRID1,GRID2,GRIK1,GRIK2,GRIK3,GRIK4,GRIN2A,GRIN2B,GRIN2C,GRM7,IL1RAPL1,KCNA5,KCNC2,KCND2,KCND3,KCNH1,KCNJ5,KCNJ6,KCNJ16,KCNK2,KCNMA1,KCNN3,KCNQ1,KCNQ3,KCNS3,TTYH2,CLIC5,PKD1L1,FXYD7,DENND5B,HCN1,KCNK10,TRPM5,NALCN,TMEM38A,TRPM8,CNGB3,PSEN1,ANO1,REST,TMEM38B,KCNIP2,KCNIP1,RYR1,RYR2,RYR3,T

				RPM3,SCN2A,SCN4B,SCN9A,TRPV6,ASIC4,CLCA4,CHRNA9,RASA3,PIEZO2,KCNIP4,SNAP25,ANO3,CACNA2D3,SCN3B,STIM1,ABCC8,ANO4,TRPC1,TRPC4,ANO5,KCNH6,KCNQ5,KCNK13,KCNK12,KCNT2,KCNAB1,ANO2,TRPC7,GLRA3,JPH2,ANO6
Pathway	substrate-specific channel activity	1.75E-04	140	CACNA1H,CACNA1A,CACNA1B,CACNA1C,CACNA1D,KCNH7,ASIC2,STIM2,ANXA6,KCNAB2,SHROOM2,AQP1,AQP5,AQP6,AQP9,ANO8,SLC26A7,MCU,HCN2,C15orf27,CACNA1E,CACNA2D1,CACNB2,CACNB4,TRPA1,CHRNA7,CHRNB2,CLCN4,CLCNKB,SLC24A2,CNGA1,CNGA3,TRPM6,PIEZO1,GRIN3A,KCNMB2,KCNK9,CACNG3,CACNG2,PEX5L,CACNG5,GABRA3,KCNH5,GABRA4,GABRA5,GABRB1,GABRB2,GABRB3,GABRG3,GABRR1,GABRR2,ASIC5,GLRA1,GLRA2,GPM6A,TMC1,TMC2,GRIA1,GRIA2,GRIA4,GRID1,GRID2,GRIK1,GRIK2,GRIK3,GRIK4,GRIN2A,GRIN2B,GRIN2C,GRM7,IL1RAPL1,KCNA5,KCNC2,KCND2,KCND3,KCNH1,KCNJ5,KCNJ6,KCNJ16,KCNK2,KCNMA1,KCNN3,KCNQ1,KCNQ3,KCNS3,TTYH2,CLIC5,PKD1L1,FXYD7,DENND5B,HCN1,KCNK10,TRPM5,NALCN,TMEM38A,TRPM8,CNGB3,PSEN1,ANO1,REST,TMEM38B,KCNIP2,KCNIP1,RYR1,RYR2,RYR3,TRPM3,SCN2A,SCN4B,SCN8A,SCN9A,SCNN1G,TRPV6,ASIC4,CLCA4,CHRNA9,RASA3,PIEZO2,KCNIP4,SNAP25,ANO3,CACNA2D3,SCN3B,STIM1,ABCC8,ANO4,TRPC1,TRPC4,ANO5,KCNH6,KCNQ5,KCNK13,KCNK12,KCNT2,KCNAB1,ANO2,TRPC7,GLRA3,JPH2,ANO6
Pathway	PDZ domain binding	4.16E-04	57	ACVR2A,APBA1,CASK,ATP2B1,ATP2B2,HCN2,LIN7A,CACNA1D,SLC9A3R2,DLGAP3,ARHGAP29,ERC2,CXADR,DOCK4,LZTS3,BDNF,MAGI2,KIF14,DLG1,DLG2,DTNA,TBC1D10A,PLEKHA2,CRIM1,NCOA2,MPP6,FZD2,IGSF5,KIRREL3,GRIA1,GRIA2,GRID2,GRIK2,GRIN2C,GRM7,EXOC4,L1CAM,LRP2,MPP2,MUSK,NSF,SNTG2,PARK2,PDZK1,PSEN1,PTEN,CCDC88C,SDC2,SFRP1,NLGN1,CXXC4,SNTA1,SNTB1,ERC1,GNG12,ADGRL2,CADM1
Pathway	cation channel activity	6.26E-04	104	SCN8A,SCN9A,KCNH7,ASIC2,STIM2,KCNAB2,SHROOM2,AQP1,MCU,HCN2,C15orf27,CACNA1H,CACNA1A,CACNA1B,CACNA1C,CACNA1D,CACNA1E,CACNA2D1,CACNB2,CACNB4,TRPA1,CHRNA7,CHRNB2,SLC24A2,CNGA1,CNGA3,TRPM6,PIEZO1,GRIN3A,KCNMB2,KCNK9,CACNG3,CACNG2,PEX5L,CACNG5,KCNH5,ASIC5,GPM6A,TMC1,TMC2,GRIK1,GRIN2A,GRIN2B,G

				RIN2C,GRM7,IL1RAPL1,KCNA5,KCNC2,KCND2,KCND3,KCNH1,KCNJ5,KCNJ6,KCNJ16,KCNK2,KCNMA1,KCNN3,KCNQ1,KCNQ3,KCNS3,PKD1L1,DENND5B,HCN1,KCNK10,TRPM5,NALCN,TMEM38A,TRPM8,CNGB3,PSEN1,ANO1,REST,TMEM38B,KCNIP2,KCNIP1,RYR1,RYR2,RYR3,TRPM3,SCN2A,SCN4B,SCNN1G,TRPV6,ASIC4,CHRNA9,RASA3,PIEZO2,KCNIP4,SNAP25,CACNA2D3,SCN3B,STIM1,ABCC8,TRPC1,TRPC4,KCNH6,KCNQ5,KCNK13,KCNK12,KCNT2,KCNAB1,TRPC7,JPH2,ANO6
Pathway	calcium ion transmembrane transporter activity	6.96E-04	51	SLC24A4,STIM2,MCU,ATP2A1,ATP2B1,ATP2B2,C15orf27,CACNA1H,CACNA1A,CACNA1B,CACNA1C,CACNA1D,CACNA1E,CACNA2D1,CACNB2,CACNB4,TRPA1,SLC24A2,TRPM6,GRIN3A,CACNG3,CACNG2,ATP2C1,CACNG5,GPM6A,TMC1,TMC2,GRIN2A,GRIN2B,GRM7,IL1RAPL1,PKD1L1,DENND5B,NALCN,TRPM8,PSEN1,RYR1,RYR2,RYR3,TRPM3,TRPV6,CHRNA9,RASA3,SLC8A1,SLC8A3,CACNA2D3,STIM1,TRPC1,TRPC4,TRPC7,JPH2
Pathway	passive transmembrane transporter activity	7.46E-04	144	KCNH7,ASIC2,STIM2,ANXA6,KCNAB2,SHROOM2,AQP1,AQP5,AQP6,AQP9,ANO8,SLC26A7,MCU,BCL2,HCN2,C15orf27,CACNA1H,CACNA1A,CACNA1B,CACNA1C,CACNA1D,CACNA1E,CACNA2D1,CACNB2,CACNB4,TRPA1,CHRNA7,CHRNB2,CLCN4,CLCNKB,SLC24A2,CNGA1,CNGA3,TRPM6,PIEZO1,GRIN3A,KCNMB2,KCNK9,CACNG3,CACNG2,PEX5L,CACNG5,GABRA3,KCNH5,GABRA4,GABRA5,GABRB1,GABRB2,GABRB3,GABRG3,GABRR1,GABRR2,ASIC5,GJA3,GLRA1,GLRA2,GJA10,GPM6A,TMC1,TMC2,GRIA1,GRIA2,GRIA4,GRID1,GRID2,GRIK1,GRIK2,GRIK3,GRIK4,GRIN2A,GRIN2B,GRIN2C,GRM7,IL1RAPL1,KCNA5,KCNC2,KCND2,KCND3,KCNH1,KCNJ5,KCNJ6,KCNJ16,KCNK2,KCNMA1,KCNN3,KCNQ1,KCNQ3,KCNS3,TTYH2,CLIC5,PKD1L1,FXYD7,DENND5B,HCN1,KCNK10,TRPM5,NALCN,TMEM38A,TRPM8,CNGB3,PSEN1,ANO1,REST,TMEM38B,KCNIP2,KCNIP1,RYR1,RYR2,RYR3,TRPM3,SCN2A,SCN4B,SCN8A,SCN9A,SCNN1G,TRPV6,ASIC4,CLCA4,CHRNA9,RASA3,PIEZO2,KCNIP4,SNAP25,ANO3,CACNA2D3,SCN3B,STIM1,ABCC8,ANO4,TRPC1,TRPC4,ANO5,KCNH6,KCNQ5,KCNK13,KCNK12,KCNT2,KCNAB1,ANO2,TRPC7,GLRA3,JPH2,GJC2,ANO6
Pathway	protein domain specific binding	2.37E-03	212	ACVR2A,AXIN1,MKL1,SORBS2,APBA1,APP,SHROOM2,DENND1A,AR,CASK,ARHGAP5,ARHGAP6,ARL1,SKAP1,IRS2,ATP1A1,ATP2B1,ATP2B

				2,CRADD,ADAM9,BCL2,HCN2,LIN7A,CFLAR,SYNJ1,CACNA1C,CACNA1D,CACNB2,RUNX2,CBL,PIAS2,LDB2,RASSF9,LRRFIP2,DLGAP2,DLGAP1,PTTG1,SLC9A3R2,DLGAP3,ARHGAP29,ABCC2,QKI,AKAP7,RPH3AL,LITAF,SCAMP1,ZNF521,CPE,CRK,NCOR1,RGS6,CTR9,ERC2,CTBP1,ARHGEF4,CTNND1,RIMS2,CXADR,DOCK4,LZTS3,MTSS1,DAB1,DNAJC6,ELMO1,GAB2,MAGI2,KIF14,VGF,DLG1,DLG2,SH3BGRL2,DNMT1,DOCK1,DPYSL3,DRD4,PDCD6IP,DTNA,TOM1L1,EBF1,TBC1D10A,PLEKHA2,OSTF1,EPHA4,EPS15,CTTNBP2,CRIM1,ESRRG,ETV6,ACSL3,DLC1,BAIAP2,NCOA2,EVL,SGIP1,SLC34A2,SARNP,KHDRBS3,KHDRBS1,CARD11,MPP6,FZD2,NLK,GABRR1,GABRR2,IGSF5,ARFIP1,GHR,KIRREL3,MYPN,GNAS,YWHAQ,GRB2,GRIA1,GRIA2,NPAS4,TWIST2,GRID2,GRIK2,GRIN2C,GRM7,FAF1,AFAP1,HDAC1,EXOC4,HIVEP1,SHANK3,MYOCD,INPP5A,IRS1,MPP7,KPNB1,L1CAM,LRP2,MAPT,MEF2C,MLLT4,MPP2,PYCARD,MUSK,MYO7A,HNRNPM,NPHS1,NSF,SNTG2,PARK2,PAX3,PAX6,PDZK1,STRN4,CARD14,STRN3,SH3KBP1,PPARA,PRKCE,PSEN1,PTEN,PTPN12,RAB6A,RAB27B,USP47,KHDRBS2,ARHGAP17,CCDC88C,ROBO1,RXRA,FMN1,SCN2A,SCNN1G,SDC2,SFRP1,SHC4,NLRP1,NLGN1,SKI,SHANK2,ENAH,CXXC4,RIMS1,SNAP25,OCLN,SNTA1,SNTB1,UNC13A,SON,SP100,ERC1,ARHGAP26,NFASC,NOD2,STRN,STX1A,STXBP1,GNG12,TACC1,ADGRL2,TCF7L2,TCF12,TFDP2,TGM2,MPP5,SH3PXD2B,TLN1,SRRM2,TP53BP2,NUP62,CADM1,UVRAG,VCP,WT1,XPO1,ZBTB16,HMGA2
Pathway	guanyl-nucleotide exchange factor activity	1.27E-02	9	ALS2,ARHGEF4,FARP2,DOCK2,FARP1,VAV3,PREX2,TIAM1,SPATA13
